# Supplementary material for: Voltage assisted asymmetric nanoscale wear on ultra-smooth diamond like carbon thin films at high sliding speeds
Source: Sci Rep. 2016 May 6;6:25439. doi: 10.1038/srep25439 (PMC4858732; doi:10.1038/srep25439)
Supplement: Supplementary Information [file srep25439-s1.pdf]

Supplementary Information:
Voltage assisted asymmetric nanoscale wear on ultra-smooth diamond like carbon thin films at high sliding speeds

Sukumar Rajauria, Erhard Schreck, and Bruno Marchon
HGST, a Western Digital Company, Recording Sub System Staging and Research, San Jose, CA 95135 USA.
(Dated: April 10, 2016)

INITIAL FLY HEIGHT DEPENDENCE ON INTERFACIAL VOLTAGE

Interfacial voltage between the head and the disk create an attractive electrostatic force which in-turn change the initial flying clearance. The electrostatic force between the head and disk could be estimated like two parallel plates, which is given by: $F_{el} = (\epsilon_0\epsilon_r A/2d^2)V^2$, where $\epsilon_0\epsilon_r$ is the dielectric permeability, A is the plate area, d is their separation and V is the potential difference between the plate. In head-disk interface there exist an intrinsic voltage difference between the head and disk surface, known as null voltage ($\sim$ 0.1-0.3 V) [1, 2]. To avoid any confusion, the interfacial voltage in this manuscript is defined with respect to null voltage.

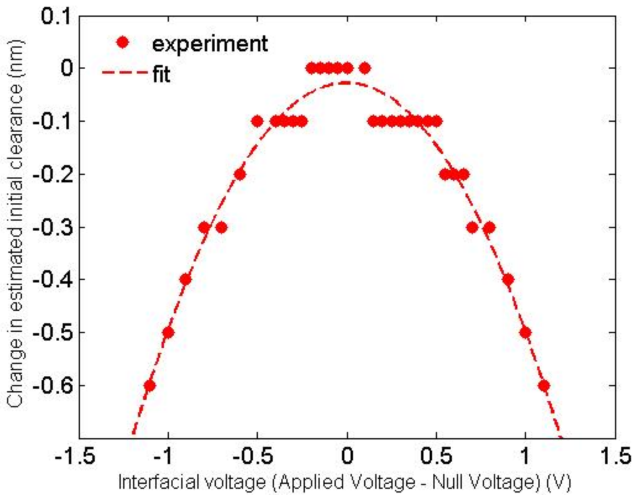

FIG. S1: Change in estimated initial clearance as a function of interfacial voltage at the head-disk interface.

Figure S1 shows the change in estimated clearance as a function of interfacial voltage at the head disk interface along with the parabolic fit. The estimated clearance change is measured by monitoring the change in heater power required to make a contact between the head and disk. It is worth-mentioning that although the change in initial flying clearance is symmetric to the interfacial voltage but the head overcoat wear is not symmetric to the interfacial voltage (see Figure 2(a)).

INTERFACIAL CURRENT DECAY AT TWO LOAD CONDITIONS

Figure S2 shows the decay in the interfacial voltage between the head and disk at two estimated normal load conditions. Interfacial current decay is similar for the two cases except that the residual current at long time is more for high load condition. This is due to load dependent area of contact which increase with load at an interface.

HEAD CARBON OVERCOAT WEAR AT TWO LOAD CONDITIONS

Figure S3(a) shows the head carbon overcoat wear rate as a function of interfacial voltage at the head and disk interface at two estimated load conditions. For green open dots the estimated load is negligible as the head and disk

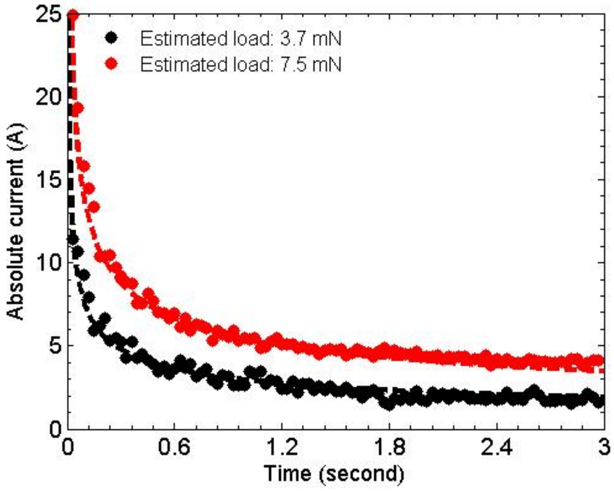

FIG. S2: Shows the decay in interfacial current decay at two estimated load conditions.

are barely in contact mode, where the head dynamics during contact is minimal. For blue dots, head is under an overpush condition and load is estimated to be around 2.5 mN. Although the absolute wear rate is different for two conditions with high load leading to higher overcoat wear rate but the overall dependence of head overcoat wear on interfacial voltage is similar with positive voltage on head leading to higher wear compare to negative bias voltage.

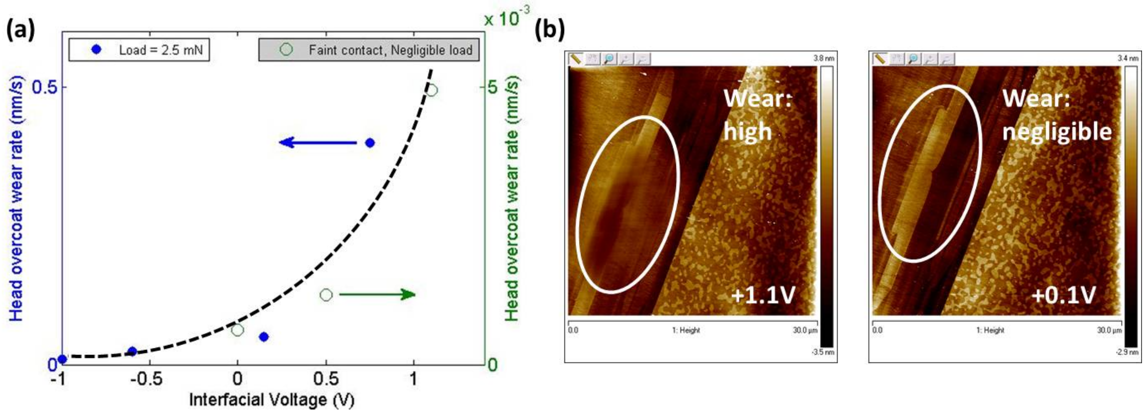

FIG. S3: (a) Shows the head overcoat wear rate as a function of interfacial bias voltage on the head overcoat at two different estimated load conditions. Green open dots correspond to a barely contact situation corresponding to a negligible load. Blue dots correspond to a normal load of 2.5 mN. (b) AFM image of two heads after a same wear cycle at two different interfacial bias voltage on the head overcoat.

Figure S3(b) shows the AFM image of two heads underwent a same wear cycle at two different interfacial voltage. The estimated normal load for two conditions are negligible with head barely in contact with the disk (touchdown). For interfacial voltage of $+1.1$ V, the head carbon overcoat wear significantly with a depth of 1 nm, and for interfacial voltage of $+0.1$ V the head overcoat wear is negligible.

Figure S4 shows the scanning electron microscope (SEM) image of two heads after a same wear cycle of negligible load (touchdown only) at two different interfacial bias voltage polarity on the head overcoat. It clearly shows that the head overcoat wear is much more for positive interfacial voltage (wear measured from Auger is 0.9 nm) in comparison to the reverse voltage polarity (wear measured from Auger is 0.1 nm).

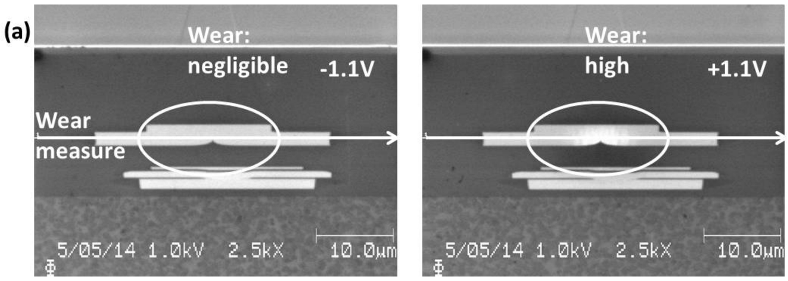

(b)

Interfacial voltage	Auger wear
-1.1 V	0.1 nm
+1.1 V	0.9 nm

FIG. S4: (a) Shows the scanning electron microscope image (SEM) image of two heads after a same wear cycle of negligible load (touchdown only) at two different interfacial bias voltage polarity on the head overcoat. (b) Table quantifying overcoat wear using Auger for the two conditions.

EXPERIMENT PROTOCOL FOR IN-SITU MONITORING OF WEAR ON HEAD OVERCOAT

Head-disk interface of the hard disk drive has a unique feature with an embedded micro-heater in the head. Micro-heater generates a localized protrusion on the head surface, thus bringing it in contact with the rotating disk. The power at which the head starts to make a contact with the disk is called touchdown power. Further increase in micro-heater power above the touchdown power, called overpush power, increases the applied normal load at the interface. The overpush power is typically applied for around 30 ms, after which micro-heater power is reduced to a value lower than touchdown power such that there is no contact between the head and disk. This overpush cycle lead to wear on head overcoat which is quantified by an increase in touchdown power (delta touchdown power) on a repeat cycle. We repeat this over many cycles and monitor the increase in micro-heater touchdown power in each cycle. Increase in micro-heater power is calibrated to estimate the wear depth in a continuous manner during the experiment.

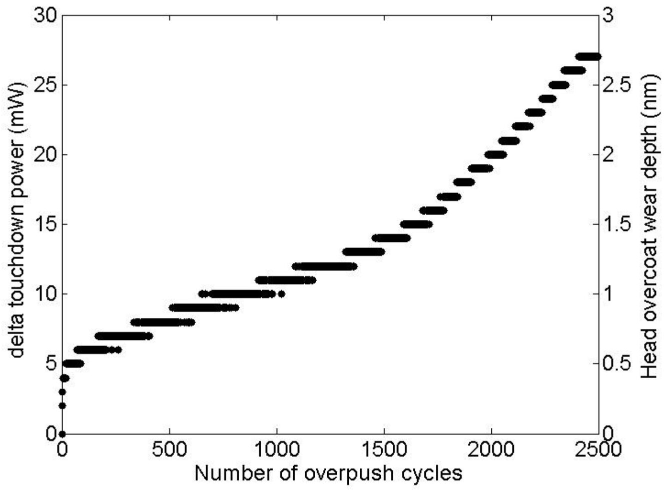

FIG. S5: Left and Right y-axis shows the delta touchdown power and the estimated head overcoat wear depth as a function of repeated number of overpush cycles.

Figure S5 shows a typical head overcoat wear experiment. Left y-axis is the delta touchdown power (or increase in micro-heater power) as a function of successive overpush cycles. Each overpush cycle creates wear on head overcoat, and therefore on a successive touchdown cycle the micro-heater requires an extra power to make a contact with the disk. This increase in touchdown power is calibrated to monitor to nano-meter level the head overcoat wear depth [3]. Right y- axis shows the corresponding estimated wear depth on the head overcoat.

[1] Baumgart, P. M., Knigge, B. and Mate, C. M. Magnetic recording disk drive with actively controlled electric potential at the head/disk interface. US Patent 7,016,138 filed 22 Oct. 2003, and issued 21 Mar. 2006.
[2] Murthy, A. N., Pit, R. and Flechsig, K. A. In-situ contact potential measurement in hard disk drives using head disk interface voltage control. *ASME 2014 Conference on Information Storage and Processing Systems* **ISPS2014-6994**, V001T01A027 (2014).
[3] Chen, Y.-K., Murthy, A. N., Pit, R. and Bogy, D. B. Angstrom scale wear of the air-bearing sliders in hard disk drives. *Tribology Letters* **54**, 273-278 (2014).
